# Medical Jurisprudence

**Published:** 1854-01

**Authors:** 


*Medical Jurisprudence.* By Alfred S. Taylor, M. D., F. R. S., etc., edited with additions, by Edward Hartshorne, M. D. etc. Philadelphia: Blanchard & Lea, 1854.

The third American from the fourth London edition of Dr. Taylor's very valuable work has been received from the publishers. There is not attached in this country the degree of interest to Medical Jurisprudence that there is in England. Here it is very seldom the case that coroners are chosen from the medical profession, and we are satisfied that there is not generally that careful scrutiny in reference to the cause of death in cases of inquest that there would be, were the examinations conducted by well educated medical men. There is more importance attached in this country to those medico-legal questions of a civil, or if criminal not of a capital nature.

The subject of poisons is treated of very fully and explicitly, the tests for the different articles usually made use of minutely and carefully described, and their effects on the system clearly and accurately pointed out.

The relation which the physician sustains to the community renders it obligatory upon him to be always prepared, not only to apply the resources of our science in relieving the suffering and restoring them to health, but also in detecting crime, bringing the offender to justice, and in assisting the proper officers in determining the moral responsibility of the insane. In order that these trusts may be met, no physician should remain ignorant in regard to those subjects on which he is liable at any day to be questioned before our legal tribunals. For the benefit of the accused, for the interest of society, and for the honor of the profession he should qualify himself to give an intelligent and correct opinion whenever his knowledge shall be required by the State or by the individual.

We have been led to these reflections from a conviction of the fact, that often the criminal has escaped unwhipt of justice, while the innocent has been punished, that the insane have been imprisoned, while those feigning insanity have been tenderly cared for in an asylum, either from the ignorance of medical witnesses or from the low estimate placed by juries and judges on medical testimony. We cannot wonder that this is the case, when we remember that our laws recognize no difference between the self-constituted doctor, the man who affixes the title to his name, but is destitute of every qualification ; and him who, laboring earnestly and patiently, has won a right to the title, and received as a sanction to his practice and a guarantee for his knowledge the approbation of medical schools, or of regularly organized medical societies. We do not insist that every man shall have a diploma, but that physicians are the proper persons to judge of the qualifications necessary to the practice of the *ars medendi.* The most miserable pretenders are paraded in our courts of justice, insulting our juries, mocking our judges, and jeopardizing the lives and liberties of our citizens by their ignorance and stupidity. Medical witnesses are too often chosen, not in consequence of their acknowledged skill and attainments, but with reference to the nature of the testimony which they are expected to give, whether favorable or otherwise. The result of all this is, that our profession is injured in the estimation of the law and of the public. The adverse opinions and conflicting testimony so frequently produced, gives rise to the idea, that in medicine there is nothing certain, that there are no fixed principles. We know of no remedy for this, unless it be for all who would claim respectability in our profession to obtain a real and not a fictitious knowledge, basing our claims, like the lawyer and the divine, upon what we are able to perform, while at the same time we avail ourselves of every legitimate means for discouraging quackery and pretension, and for diffusing among the people that amount of medical knowledge which will prepare them, not to be their own doctors, but to exercise a discrimination in their choice of physicians to enable them to recognise and appreciate real attainments.

But we have wandered away from Dr. Taylor's book, which by the way we would recommend to all who are desirous of becoming well prepared for this part of professional duty.

For sale by D. B. Cooke & Co., 135 Lake st. J.

